# Targeting senescent cells: approaches, opportunities, challenges

**DOI:** 10.18632/aging.102557

**Published:** 2019-11-30

**Authors:** Cayetano von Kobbe

**Affiliations:** 1 Centro de Biología Molecular “Severo Ochoa” (CBMSO), Consejo Superior de Investigaciones Científicas (CSIC), Universidad Autónoma de Madrid, Madrid 28049, Spain

**Keywords:** cellular senescence, senolytics, senomorphics, immunosurveillance, anti-aging therapies

## Abstract

Cellular senescence is a hallmark of aging, whose onset is linked to a series of both cell and non-cell autonomous processes, leading to several consequences for the organism. To date, several senescence routes have been identified, which play a fundamental role in development, tumor suppression and aging, among other processes. The positive and/or negative effects of senescent cells are directly related to the time that they remain in the organism. Short-term (acute) senescent cells are associated with positive effects; once they have executed their actions, immune cells are recruited to remove them. In contrast, long-term (chronic) senescent cells are associated with disease; they secrete pro-inflammatory and pro-tumorigenic factors in a state known as senescence-associated secretory phenotype (SASP). In recent years, cellular senescence has become the center of attention for the treatment of aging-related diseases. Current therapies are focused on elimination of senescent cell functions in three main ways: i) use of senolytics; ii) inhibition of SASP; and iii) improvement of immune system functions against senescent cells (immunosurveillance). In addition, some anti-cancer therapies are based on the induction of senescence in tumor cells. However, these senescent-like cancer cells must be subsequently cleared to avoid a chronic pro-tumorigenic state. Here is a summary of different scenarios, depending on the therapy used, with a discussion of the pros and cons of each scenario.

## INTRODUCTION

Cellular senescence is a stress response mechanism induced by different types of insults such as telomere attrition, DNA damage, and oncogenic mutations, among others [1]. First described in cultured human diploid fibroblasts after successive rounds of division [2], its main hallmarks are irreversible growth arrest, alterations of cell size and morphology, increased lysosomal activity, expression of anti-proliferative proteins, resistance to apoptosis, activation of damage-sensing signaling routes. Another important characteristic is the regulated secretion of interleukins (ILs), inflammatory factors, chemokines, proteases and growth factors, termed the senescence-associated secretory phenotype (SASP) [3].

As there is ample evidence placing senescent cells as one of the causes of age-related dysfunctions, it has been considered to be one of the hallmarks of aging [4]. It was recently demonstrated that elimination of senescent cells by genetic or pharmacological approaches delays the onset of aging-related diseases, such as cancer, neurodegenerative disorders or cardiovascular diseases, among others, showing that the chronic presence of these cells is not essential [5–7]. Conversely, local injections of senescent cells drive aging-related diseases [8, 9]. This data, together with that obtained from tissues of patients with different diseases and ages, has established causality of senescent cells in some aging-related pathologies [10, 11].

Current therapies targeting senescent cells are focused on: i) specific killing of these cells by senolytics; ii) specific inhibition of the secretory phenotype (anti-SASP strategy); and iii) improving clearance of senescent cells by the immune system [12]. In addition, currently available senescence-inducing therapies for cancer stop tumor growth while causing accumulation of senescent cells [13, 14], which subsequently become a problem for the organism [15].

This review will summarize the hypothetical scenarios that each anti-cell senescence approach (described above) could face, either alone or in combination, with a discussion of open questions that should be kept in mind when targeting senescent cells.

### Triggers of cell senescence

The onset of senescence in healthy tissue occurs in response to different internal and external stimuli, such as telomere attrition, DNA damage (alkylating agents, radiation), oncogene activation, mitochondrial dysfunction, and spindle, epigenetic, endoplasmic reticulum (ER) and proteotoxic stress [16–19]. The type and duration of the stimulus dictates the final effect on the senescent cells [20]. These cells display a characteristic phenotype comprising specific cell/nuclear morphology (increased size, abnormal shape and nuclear envelope changes), apoptosis resistance, chromatin redistribution (senescence-associated heterochromatin foci and senescence-associated distension of satellites), epigenetic markers (e.g. H3K9Me3), lipofuscin accumulation, SASP, and overexpression of proteins such as p53, p16^Ink4a^, p21^WAF1^, Differentiated Embryo Chondrocyte-expressed gene 1 (DEC1) and senescence-associated β-Gal (SA-β-Gal) [13, 21–24]. To date there is no universal marker for senescence, and identification of senescent cells is based on the combined detection of two or more phenotypic aspects mentioned above, such as SA-β-Gal, p16^Ink4a^ or p21^WAF1^ [10].

**Figure 1:**
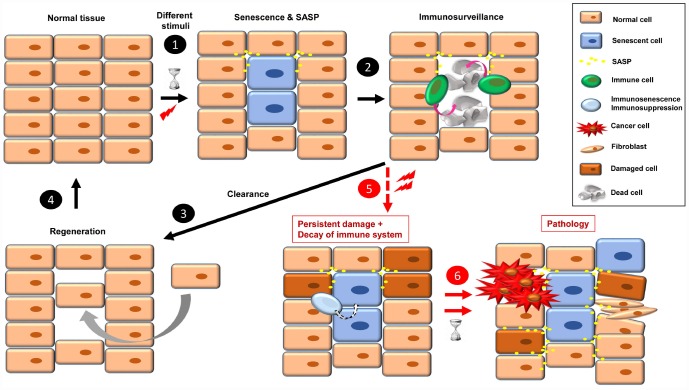
The onset of cellular senescence in normal tissue takes place in response to different stimuli (**1**). Some SASP factors are involved in immune cell recruitment, which act in the clearance of the senescent cells (**2**). Then, to restore the normal tissue, a regeneration process is necessary (**3**, **4**). When a combination of persistent damage and immune system decay occurs, senescent cells accumulate, creating a pro-inflammatory and pro-tumorigenic environment and fibrotic tissue. Over time, this leads to disease, such as cancer progression, insulin resistance, osteoarthritis, atherosclerosis, and brain pathologies, among others (**5**, **6**).

One of the characteristic phenotypic hallmarks of cell senescence is the secretion of a plethora of factors that affect their environment (SASP), which also serves as a call for the immune system to recognize and eliminate the senescent cells [3, 25]. Among the SASP factors that seem responsible for attraction of immune cells are CSF (colony stimulating factor 1), CXCL-1 (chemokine C-X-C motif ligand 1), MCP-1 (monocyte chemoattractant protein 1) and ICAM-1 (intercellular adhesion molecule 1) [25]. In this scenario of acute or short-term senescence, the tissue returns to normal after a regeneration process [17] (Figure 1, steps 1-4). The regeneration is a fundamental process to avoid tissue atrophy and dysfunction. In this scenario of replacement of senescent cells, we should keep in mind the different capacity of renewal of some tissues with respect to others, and the exhausted or damaged state of stem cells that can lead to functionally compromised differentiated cells or carcinogenesis [26].

### Implication of cell senescence in disease

Acute senescent cells play a direct role in tumor suppression, efficient wound healing, embryogenesis, placental formation, and tissue regeneration, among other processes [17]. At this point, both their onset and primary effect are positive for the organism [17, 20].

When senescence-inducing stimuli persist and decrease the ability of the immune system to recognize and eliminate senescent cells (by either immunosenescence or immunosuppression), these cells accumulate. The continual presence of senescent cells negatively affects their environment, inducing damage, instability or senescence in other cells through SASP [1, 27]. Over time, these “secondary” damaged cells can become either pro-tumorigenic or senescent, which increases the cellular instability of the tissue, leading to dysfunction and disease [27] (Figure 1, steps 5 and 6). In this sense, some SASP factors play a direct role in fibroblast activation and uncontrolled fibrotic scarring [28].

Chronic senescent cells (also termed “zombie” cells) have been associated with the onset of several diseases [1, 10, 13, 17]. In the last few years there have been extensive studies to elucidate the causative role of senescence in the onset of different pathologies [17]. These studies were mainly based on: i) detection of senescent cells in tissues/organs from patients or animal models; or ii) improvement in tissue/organ functions upon removal of senescent cells in mice, by either genetic or pharmacological interventions. This is a list of some age-related diseases where cellular senescence seems to play an important role:

### Cancer

Aging is the main cause of cancer [29], and the presence of senescent cells in aged tissues or xenograft models correlates with the incidence of cancer [30, 31]. Their specific removal led to a delay in tumor formation and reduced metastasis [6]. It is also important to note that both senolytics and senomorphics are currently being used in clinical trials for the treatment of numerous types of cancer, such as leukemia, lung cancer, melanoma and glioblastoma, among others [16].

### Neurodegenerative disorders

Senescent cell accumulation has been detected at sites of brain pathology [7, 32, 33]. The presence of senescent astrocytes correlates with the onset of pathologies such as Parkinson’s and Alzheimer’s disease [34]. Interestingly, Tau protein induces cellular senescence in neurons, and specific clearance of senescent astrocytes and microglia, reduced Tau-containing neurofibrillary tangle, neuron loss and ventricular enlargement [7, 8]. Moreover, it has been proposed a role of senescent cells in multiple sclerosis [33].

### Cardiovascular disease

Senescent cells play a key role in atherosclerosis, and their specific removal reduced progression of the disease [35]. Moreover, senescent macrophages seem to contribute to coronary heart disease, and cell senescence in the aorta increases vascular stiffness [13].

### Osteoarthritis

This degenerative disease causes the joints to become painful and stiff, and accumulation of senescent cells correlates with its progression [36]. In mouse models, local injections of these cells induce an osteoarthritis-like condition [9], whereas their clearance improves health by attenuating development of post-traumatic osteoarthritis [37].

### Type 2 diabetes

Aging is the main cause of type 2 diabetes, and there is association between disease progression and detection of senescent markers. Senescent β-cells affect glucose homeostasis, although further work is needed to elucidate the exact role of senescence [20, 38, 39].

### Kidney-related diseases

Diseases such as glomerulosclerosis and nephropathies are associated with an increase of senescent cells [10]. Remarkably, when these cells were removed by genetic approaches, kidney functions improved [6].

### Idiopathic pulmonary fibrosis (IPF)

This chronic lung disease results in scarring, affecting primarily older adults. Tissues from IPF patients display some phenotypical characteristics of senescent cells, and when these cells were removed by senolytics, pulmonary functions improved [104].

### Cachexia

In this disease adipocyte differentiation is disrupted by senescent cells, causing weight loss, muscle wasting and loss of body fat, leading to metabolic dysfunction and loss of adaptive thermogenic capacity [10]. When senescent cells were removed, tissue homeostasis recovered [6, 75].

### Cataracts

Characterized by opacity of the lens of the eye [109], the lens capsules from patients suffering cataracts show accumulation of senescent human lens epithelial cells [105]. Removal of these cells by genetic approaches decreased the incidence of cataracts in old mice [6].

### Liver diseases

The presence of senescent cells correlates with the onset of liver fibrosis, cirrhosis and non-alcoholic fatty liver disease. Elimination of these cells reduced liver fat accumulation [10, 106].

### Metabolic syndrome

A collection of metabolic disorders such as increased blood pressure, high blood sugar, excess body fat (around the waist) and abnormal cholesterol levels. Endothelial cell senescence is involved in systemic metabolic dysfunction and glucose intolerance [13, 107].

### Erectile dysfunction

The presence of senescent cells is directly related to endothelial dysfunction. SASP factors seem mediate this effect, and importantly, removal of senescent cells led to improvement of erectile function in mice [40].

Altogether, this data highlights the importance of targeting these cells in order to delay or cure different diseases.

## STRATEGIES TO SUPPRESS SENESCENT CELLS

### Senolytics

**Figure 2:**
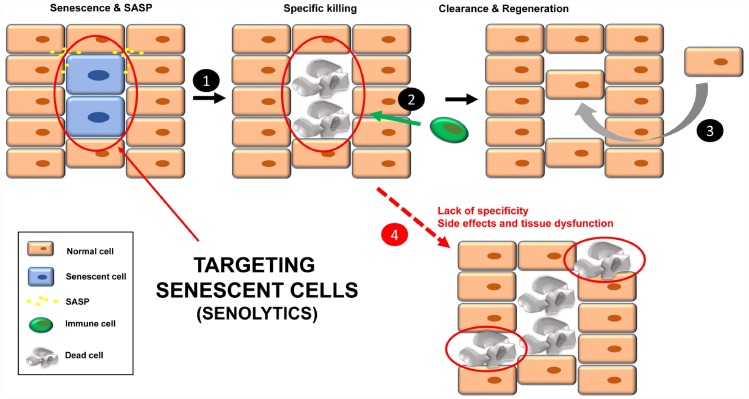
Treatment with senolytics to specifically kill senescent cells (**1**). Over time, these apoptotic bodies will be cleared by the immune system (**2**). Finally, a regenerative process will lead to normal tissue functions (**3**). Normal cells could be affected by either the lack of specificity of the senolytics or chronic treatment, leading to tissue dysfunction (**4**).

An option to eliminate the negative effects of chronic senescent cells is to kill them specifically, using compounds called senolytics (Figure 2), which target pathways activated in senescent cells [16]. The list of these senolytic tool compounds is extensive and continuously growing. In Table 1 are shown the noteworthy ones. Chronic/periodic administration of senolytics kills senescent cells that are generated in the tissues, and the immune system is responsible for clearing apoptotic bodies for subsequent regeneration with new cells (Figure 2, steps 1-3). Senolytics target key proteins mainly involved in apoptosis, such as Bcl-2, Bcl-X_L_, p53, p21, PI3K, AKT, FOXO4 and p53. See Table 1 for references.

**Table 1:** List of senolytics and their targets.

**Senolytic**	**Target/function**	**References**
	Apoptosis	
Dasatinib (D)	Inhibitor EFNB*-dependent suppression of apoptosis	[51]
Quercetin (Q)	PI3K/AKT, BCL-2, p53, p21, Serpine	[51]
ABT 737	BCL-W and BCL-XL inhibitor	[52]
ABT 263 (Navitoclax; UBX0101)	BCL-2, BCL-XL and BCL-W inhibitors	[37, 53, 54]
A1331852, A1155463	BCL-XL	[55]
Fisetin	PI3K/AKT and ROS	[55]
FOXO4-related peptide (DRI)	Inhibitor of FOXO4-p53 interaction	[44]
Delivery options**		
Gal-encapsulated cytotoxics	SA-β-Gal	[42]

*AKT; protein kinase B. BCL; B-cell lymphoma. EFNB; ephrin ligand B. FOXO; forkhead box proteins O. PI3K; phosphatidylinositol 3-kinase. ROS; reactive oxygen species.

**It helps improve senolysis by directed targeting.

Although senolytics are supposed to be specific for senescent cells, there are always unwanted damage/side effects since the administration is not directed [41] (Figure 2, step 4). In this regard, a new strategy has been recently described to specifically target senescent cells in mice, using nanocapsules containing toxins (or senolytics) [42]. The outer layer of these nanocapsules are composed of substrates for enzymes that are overexpressed in senescent cells. In this way, the toxin (senolytic) will only be released inside senescent cells, killing them [42]. Thus, these nanocapsules are a vehicle to specifically deliver any type of senolytic into senescent cells in mice. The specificity of the delivery is important in non-targeted senolytics (natural product derivatives with less defined biological activities), such as quercetin and fisetin.

Though there have been numerous reports showing the benefits of senolytics, it is important to highlight the recently described effects of dasatinib + quercetin (D + Q) treatment on lifespan in old animals [43]. Transplant of senescent cells into healthy mice caused physical dysfunction, which was reversed by oral administration of D + Q [43]. Also, clearance of senescent neurons improved neurological functions in transgenic mice mimicking Tau aggregation-dependent neurodegenerative disease [8]. It is also important to note that the treatment with the peptide FOXO4-DRI restored renal functions in both old (normal) mice and mice with accelerated aging [44]. As indicated above, some senolytics are currently being used in clinical trials for treating different diseases [16]. In this sense it is important to mention that MDM2 inhibitors, targeting p53, are also in clinical phases as anti-cancer therapies [45].

### Remaining questions

There is reasonable doubt about the fate of the dead senescent cells, especially when the immune system of the patient is depressed (by either immunosenescence or immunosuppression). The accumulation of these apoptotic bodies may have undesired side effects (i.e. further release pro-inflammatory factors in an already-damaged tissue) [10]. Also, as indicated before, the possible side effects of periodic/chronic treatments should not be ignored. In fact, toxic effects after systemic administration of BCL family inhibitors have been described in patients, such as thrombocytopenia and neutropenia [41]. It would be desirable that treatments with senolytics are as sporadic as possible, without affecting efficacy. Lastly, and as indicated above, the regeneration process is an important issue to be analyzed in the tissues where senescence clearance has taken place.

### SASP inhibitors (or senomorphics)

**Figure 3:**
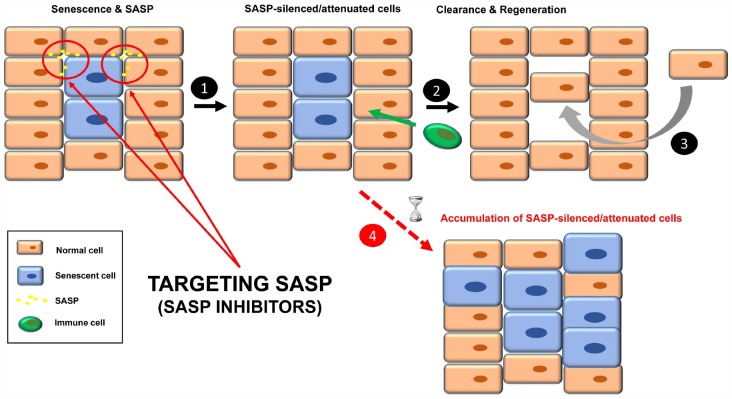
Treatment with senomorphics to inhibit SASP factors in senescent cells (**1**). Over time, these cells will be removed by immune cells (**2**). Finally, a regenerative process will lead to normal tissue functions (**3**). In aged or immunosuppressed individuals, this strategy would lead to an accumulation of SASP-silenced/attenuated senescent cells (**4**).

Another strategy to inhibit the functions of senescent cells is through the specific silencing of SASP [16, 46], the complex mixture of soluble factors such as cytokines, chemokines, growth factors, proteases and angiogenic factors that mediates the paracrine and autocrine functions of senescent cells [3, 25] (Figure 3). The qualitative and quantitative composition of this secretome is different depending on the cell type and the senescence-inducing stimulus, and becomes fully active a few days after the persistent stimulus [3, 47, 48]. Senomorphics inhibit SASP functions by targeting pathways such as p38 mitogen-activated protein kinase (MAPK), NF-κB, IL-1α, mTOR and PI3K/AKT (Table 2), which act at the level of transcription, translation or mRNA stabilization [21]. Alternatively, inhibition may be achieved by specific neutralizing antibodies against individual SASP factors (protein function inhibition), as is the case for IL-1α, IL-8 and IL-6.

**Table 2:** List of senomorphics and their targets.

**SASP inhibitor**	Target/function*	**References**
SB 203580	p38 MAPK** inhibitor	([60] Reviewed by [12])
UR-135756, BIRB 796	p38 MAPK inhibitor	[61]
Resveratrol	NF-ƙB inhibitor (IĸB-kinase inhibitor), AMPK and SIRT1 activator, others	[62–64]
Apigenin, Wogonin, Kaempferol	NF-ƙB inhibitors (IĸB-zeta)	[65]
Metformin	Inhibition of IKK/NF-ƙB, mitochondrial electron tranport, mitochondrial GPDH, and KDM6A/UTX, AMPK activator, others	[66–70]
Cortisol/corticosterone	IL-1α/NF-ƙB pathway inhibitors	[71]
NDGA	ROS (free radical scavenger)	[72]
Rapamycin	mTOR inhibitor, membrane-bound IL-1A translation inhibition, prelamin A, 53BP1	[73] [74] [110]
Ruxolitinib	Inhibition of JAK1/2 and ROCK	[75, 76]

*For many of the SASP inhibitors listed there have been described several targets.

**53BP1; p53 binding protein 1. AMPK; AMP-activated protein kinase. IKK; IĸB kinase. JAK; Janus kinase. KDM6A/UTX; lysine demethylase 6A. MAPK; mitogen-activated protein kinase. mTOR; mammalian target of rapamycin. NDGA; nordihydroguaiaretic acid. NF-ƙB; nuclear factor kappa light chain enhancer of activated B cells. ROS; reactive oxygen species.

As IL-1α plays a direct role in SASP regulation, targeting either the receptor (IL-1αR) or the ligand (IL-1α) leads to decreased global SASP expression, with special emphasis on oncogene-induced senescence (OIS) [49, 50].

Importantly, the MABp1 antibody (neutralizing anti-human IL-1α monoclonal) has proven efficient in clinical trials against type 2 diabetes, sarcopenia and inflammation [56–58], diseases in which senescent cells play an important causative role [10].

IL-8 is a member of the CXC motif chemokine upregulated in SASP, and is associated with some types of cancer [50]. ABX-IL-8 is a humanized monoclonal antibody against IL-8 that acts as an antagonist, impairing IL-8 signaling. Treatment with ABX-IL-8 attenuates the growth of some cancer xenografts models [59].

IL-6 is a pleiotropic cytokine also upregulated in SASP that is involved in tumor proliferation, invasion and immunosuppression. Specific inhibition of IL-6 by a neutralizing monoclonal antibody (Mab-IL-6.8) completely abolished JAK/STAT signaling [50, 77] and relieved symptoms of arthritis in a primate model (Olokizumab) [78]. Arthritis has also been causally associated with the presence of senescent cells [37].

Finally, SASP-silenced/attenuated senescent cells should be recognized by the immune system for subsequent clearance and regeneration (Figure 3, steps 2 and 3).

### Remaining questions

One doubt about this strategy is how SASP-silenced/attenuated senescent cells would be cleared. Given that some SASP factors are involved in the recruitment of immune cells, SASP inhibition could make senescent cells effectively “invisible” to the immune system, therefore remaining chronically within the tissue. In fact, two senomorphics (apigenin and kaempferol) showed inhibition in cultured cells of SASP components involved in immune cell recruitment, such as CXCL-1 and CSF [65]. What would the influence of SASP-silenced senescent cells be in the tissue? Perhaps instead of being dysfunctional, the tissue would be non-functional.

Likewise, as senomorphics require chronic/continuous treatment, a major problem of these types of SASP inhibitors is the lack of specificity for senescent cells. Perhaps inhibition of individual SASP components by neutralizing antibodies (as described above) would minimize the potential side effects. As indicated for senolytics, it would be desirable if over time, the treatments with senomorphics were as sporadic as possible without affecting efficacy.

### Improving immune system function

**Figure 4:**
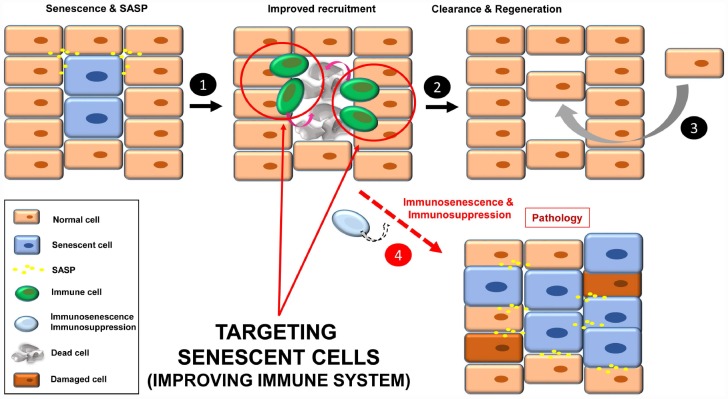
Improving immune system functions to efficiently remove senescent cells (**1**). A robust immune system targets senescent cells, leading to their removal (**2**). Then a regenerative process will maintain normal tissue functions (**3**). In situations where the immune system decays (e.g. immunosenescence or immunodepression), there will be an accumulation of senescent cells, increasing instability in the tissue/organ (**4**).

A third strategy to target senescent cells is to strengthen the immune system for efficient recognition and elimination of these cells, a process termed immunosurveillance (Figure 4, steps 1-3). The role of the immune system in the elimination of senescent cells is fundamental, and a decline in immune function is associated with an increase in the number of senescent cells and finally, disease (Figure 4, step 4) [12, 20, 79, 80].

In this regard, there are two strategies: i) improving the specific anti-senescent cell functions; and ii) general enhancement of immune functions (to avoid senescence of immune cells involved in recognition of senescent cells).

Anti-senescent cell functions have been described in NK cells, macrophages and CD4^+^ T cells [20, 81]. Since these functions take place through membrane receptors, one option is to increase the binding affinity of the involved receptors. In this sense, the use of chimeric antigen receptor (CAR) T cells to target specific senescent-related molecules would be an attractive approach. This strategy is currently showing extraordinary results as anti-cancer therapy [82]. Alternatively, specifically increasing the surface expression of these receptors in senescent cells could be attempted. NK cells recognize the CD58/ICAM1 receptor present in senescent cells [83]. In the case of macrophages this recognition is not clear, and may occur through modified membrane receptors in senescent cells (glycans, lipids or vimentin), recognized by receptors present in macrophages such as CD36, IgM, SIRPα, and leptins. For T cells this process would be mainly mediated by TCRs [84].

Another possibility is to reduce the number of senescent immune cells, perhaps by depletion using specific antibodies recognizing surface markers of senescence, and in this way “rejuvenate” the immune system [84]. In this sense the recent identification of a targetable senescent cell surface marker supports this strategy [85].

NK and T cell functions decrease in older individuals. The constitutive activation of the nutrient-sensing component adenosine 5´-monophosphate-activated protein kinase (AMPK) seems to play a central role in this process [86]. Thus, an alternative approach to increase functions of these immune cells is to target AMPK functions, as the p38 MAPK inhibitor does [87]. Another approach would be to inhibit the killer cell lectin-like receptor G1 (KLRG1, or CD57 in humans), which increases on NK and T cells of older individuals. Activation of KLRG1 in NK cells is associated with activation of AMPK (via protein stabilization), which in turn would inhibit cell functions. In the case of CD8^+^ T cells, this mechanism may involve other inhibitory receptors, such as programmed death 1 (PD-1) and cytotoxic T lymphocyte antigen 4 (CTLA-4) [86].

The down-regulation of the CD28 receptor is a hallmark of human CD8+ T cell senescence. Interestingly these senescent T cells have been found not only in old individuals (aging process), but also associated to diseases such as cancer and arthrosis [83], which are aging-related diseases where senescent cells seem to play a causative role, as discussed above.

This fact reinforces the idea of a pivotal role of immune cells by delaying the onset of diseases related to the accumulation of chronic senescent cells. In this regard, a recent article shows that mice lacking the main cytotoxic functions of NK and T cells (perforin pathway), accelerates both senescent cell burden and aging [80].

Some current anti-cancer therapies are based on immunotherapy, that stimulates the immune system to recognize and kill disease-associated cells based on differences in the expression of antigens between pathogenic and normal cells [88]. Immunotherapy is currently used not only for different types of cancer, but also for infectious diseases, Alzheimer’s disease, and even some types of addictions [89, 90]. Senescent cells display a characteristic phenotype, which make them suitable targets for this strategy. Cell and antibody mediated responses are possible approaches, however, the specificity of senescent antigens would be the bottleneck to avoid undesirable side effects [108].

### Remaining questions

Improving immune system functions to target senescent cells could be difficult in scenarios such as immunosenescence (in older individuals or patients suffering from premature aging of the immune system [91]) or immunosuppression (i.e. patients treated with corticosteroids or radiation, in cases of organ transplant, autoimmune disease or cancer). CAR-based strategies and immune system “rejuvenation” would be personalized treatments, and thus very time consuming and expensive. These strategies would rely on specific (universal) senescence receptors, and a limiting factor when detecting cell senescence is the lack of universal markers [13]. Although novel technologies are making detection of senescent cells in tissues more reliable [92, 93], the use of a combination of different biomarkers is still necessary for confirmation. Thus, personalized treatment targeting at least 2 senescence markers would increase the challenge and difficulty of the process.

Moreover, the described connection between NK and T cell activation and nutrient-sensing machinery suggests that dietary interventions could be a promising approach to maintain a healthy immune system in older individuals, and thus the ability to efficiently clear senescent cells. The up-regulation of CD28 (by forced expression of either the receptor itself or other receptor related to T cell activation) could be another attractive approach to delay the senescence process in CD8+ T cells. Last, but not least, it is important to keep in mind that a general stimulation of the components of the immune system might also induce autoimmune diseases or may also promote some hematopoietic malignancies [94, 95].

### Targeting senescent cancer cells

A way to stop cancer progression is to induce senescence in tumor cells (TIS; therapy-induced senescence), through treatments targeting key pathways activated in highly proliferative cells. These treatments include DNA damage inducers (e.g. mitoxantrone, doxorubicin, γ-radiation), and inhibitors of Aurora kinase A (i.e. MLN8054, alisertib) and CDK4/6 (abemaciclib, palbociclib, ribociclib), among others [14, 96–98]. While stopping tumor growth, TIS becomes a problem for the organism in the long-term, as cancer survivors have a higher incidence of age-related diseases linked to senescence, including cardiovascular disease, neurodegeneration, sarcopenia and secondary neoplasia [19]. Cancer cells that escape from TIS (or “senescence-like” cancer cells) display some features, such as polyploidy, stemness and aggressiveness. It has been calculated that only 1 in 10^6^ of senescent cancer cells escape from TIS. Although it seems to be a rare event, it occurs [99, 100].

**Figure 5:**
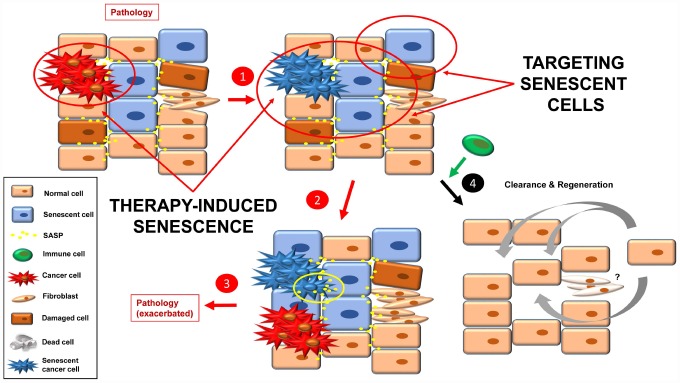
Inducing senescence in tumor cells will lead to an accumulation of senescence burden (**1**). The pro-inflammatory and pro-tumorigenic environment (more SASP factors) leads to exacerbation of the pathology (e.g. cancer relapse, fibrosis, inflammation) (**2**, **3**). By targeting senescent cells with a combination of the approaches currently used, a better final scenario is possible (**4**). Fibrotic scarring may be treated by other means, or cured over time.

At this point, it is conceivable to imagine a tissue that is already damaged, not only by tumor cells but also a mix of pre-tumorigenic and senescent cells, together with fibrosis and SASP (Figure 5). The newly senescent cells (from the tumor; TIS) would increase the level of SASP in the tissue, leading to: i) growth of new tumors (or sprouts of the former); ii) senescence induction in neighboring cells; as well as iii) an increase in fibrotic tissue. This scenario would lead to an exacerbation of the pathology that was described in the starting point (step 3).

One solution to this situation would be to combine TIS (effective therapy to stop the growth of the tumor that is already present) with one or more of the three anti-senescent strategies presented above (senolytics, senomorphics and improved immune function) (Figure 5). Then clearance and tissue renewal processes will be necessary to restore tissue functions (Figure 5, step 4).

### Remaining questions

Importantly these therapies would rely on the state of the patient´s immune system, and many patients have been affected by treatments they have received previously (immunosuppression), or by age (immunosenescence). In this sense, it is likely that in some cases it would only be necessary to inhibit SASP and not specifically induce death of the senescent cells, to avoid depending on the immune system for removal of apoptotic bodies.

And what about fibrosis? Fibrotic scarring can resolve over time, being replaced by new tissue. However, if this process is not completed (e.g. older people), the normal function of key organs can be compromised. Thus, alternative therapies should be kept in mind to treat senescence-associated fibrosis [101].

## CONCLUDING REMARKS

Targeting senescent cells has become an alternative therapy for treating different aging-related diseases. This therapy can be approached on three levels: i) specific killing of these cells; ii) inhibition of their secretory phenotype, therefore making them less efficient; and iii) improving our immune system for elimination of senescent cells.

The use of senolytics and senomorphics are showing promising results, although is still too early to draw conclusions. It is necessary to improve the specificity of these compounds, as well as optimize the treatment (i.e. dosage) to avoid unwanted effects. In this sense, progress has been made on the specific delivery of drugs into senescent cells by using nanocapsules. This elegant approach may overcome the problem of specificity of senolytic tool compounds when administrated in a chronic manner [42]. Importantly, senolytics and senomorphics are found in natural compounds, showing new (nutraceutical) approaches to treat aging-related diseases, although in a non-targeted way [102].

The “transformation” of normal cells into senescent ones is accomplished by a multitude of internal and external stressors in different physiological situations. Cancer cells can become senescent as well after different therapies, though the new tumor-induced senescent cells (TIS) generated are harmful in the long term. In this scenario, the three options presented here to either eliminate or “silence” the senescent cells are important to combat TIS. The combination of these pro- and anti-senescence approaches (TIS + senolytics and/or senomorphics and/or improved immune system), will play an important role in the cure of some types of cancer [98].

In future clinical trials focused on eliminating senescent cells, it will be important to determine when to initiate the treatments (age of the patients), the schedule (continuous, periodic and/or sporadic), as well as the specific markers to determine the efficacy of the therapy (see Table 3 for comparison of the therapies presented in this review). Clinical trials should be supported by robust preclinical results obtained in proper animal models.

**Table 3:** Comparison of the therapies presented in this review.

**Therapy**	**Pros**	**Cons**
Senolytics	High specificity - Targeted drugs Sporadic treatments - Depending on compound efficacy	Low specificity - Non-targeted compounds Side effects - BCL family inhibitors Increase in apoptotic bodies Chronic treatments - Depending on compound efficacy
Senomorphics	High specificity - Targeting individual SASP components Sporadic treatments - Depending on compound efficacy	Low specificity - Targeting central pathways Chronic treatments - Depending on compound efficacy Lack of senescent cell clearance? Side effects
Improving immune system	High specificity - Personalized treatments - Immunotherapy Sporadic treatments Dietary interventions	Time consuming and expensive - Personalized treatments Low specificity - General activation Side effects - Autoimmunity? - Hematopoietic malignancies? Chronic treatments Patients affected by immunosuppression and/or immunosenescence
TIS	High specificity - Specific targets Sporadic treatments Stops tumor growth Possibility to combine with other therapies	Low specificity - General damage (chemo-radiotherapies) Side effects - New tumors - Fibrosis Chronic treatments Increasing senescence burden

Senescent cells are the cause of several age-related diseases, which account for a high percentage of all causes of death worldwide and an expansion of morbidity. Likewise, it is estimated that by the year 2045, the number of people older than 60 will surpass, for the first time in history, the number of people under the age of 15 [103]. Thus, the approaches presented in this review highlight the urgent need for new therapies to delay or cure age/senescence-related diseases.
